# Molecular Phylogeny and Ontogeny of a New Ciliate Genus, *Paracladotricha salina* n. g., n. sp. (Ciliophora, Hypotrichia)

**DOI:** 10.1111/jeu.12117

**Published:** 2014-07-04

**Authors:** Chen Shao, Liqiong Li, Qianqian Zhang, Weibo Song, Helmut Berger

**Affiliations:** a The Key Laboratory of Biomedical Information Engineering, Ministry of Education, School of Life Science and Technology, Xi'an Jiaotong UniversityXi'an, 710049, China; b School of Ocean Science, Yantai Academe, China Agricultural UniversityYantai, 264670, China; c Yantai Institute of Coastal Zone Research, Chinese Academy of ScienceYantai, 264003, China; d Laboratory of Protozoology, Institute of Evolution and Marine Biodiversity, Ocean University of ChinaQingdao, 266003, China; e Consulting Engineering Office for Ecology5020, Salzburg, Austria

**Keywords:** *Cladotricha*, infraciliature, morphology, morphogenesis, Stichotrichida

## Abstract

A hypotrichous ciliate, *Paracladotricha salina* n. g., n. sp., was discovered in hypersaline waters (salinity about 80‰) from Qingdao, China. Its morphology and some major ontogenetic stages were studied and the phylogenetic position was estimated using standard methods. *Paracladotricha salina* is characterized by a flexible, more or less slender body (size 50–120 × 20–35 μm), a gonostomatid oral apparatus, one short and two long frontoventral rows, four macronuclear nodules, almost completely reduced dorsal kineties 1–3, and a loss of several parts of the ciliature, namely, the slightly shortened ciliary row of the adoral membranelles, the paroral, and the buccal, the postoral and pretransverse ventral, the transverse, and the caudal cirri. The ontogenesis is rather simple: anlage II of both filial products and anlage III of the opisthe originate de novo, while anlagen IV and V are formed within the parental rows. This combination of features requires the establishment of a new genus, *Paracladotricha*, which is, according to the morphological data, closely related to *Schmidingerothrix* and *Cladotricha*. The small-subunit rRNA gene was sequenced, indicating that *P. salina* is, as also demonstrated by the oral apparatus, a member of the gonostomatids. We provide a first, vague hypothesis about the phylogenetic relationships of the Gonostomatidae, Cladotrichidae, and Schmidingerotrichidae. However, since molecular data of the type species of these higher taxa are lacking, their validity and relationships remain obscure.

CLADOTRICHIDS are a relatively small and little known group of halophilous, hypotrichous ciliates. *Cladotricha koltzowii*, the type of the whole group, was discovered by Gaievskaïa (1925) in a saline lake on the Crimean Peninsula, Ukraine. Since then eight species have been described by Ruinen (1938), Blatterer and Foissner (1988), and Wilbert (1995) from saline water and soil in Australia, Indonesia, and Europe (Berger 2001). Just recently, Berger (2011) revised these species and transferred some of them to *Apourosomoida* Foissner, Agatha and Berger, 2002 because their cirral pattern deviates distinctly from that of *C. koltzowii*. Small and Lynn (1985) established Cladotrichidae comprising, besides the type genus, *Engelmanniella* Foissner, 1982, *Uroleptoides* Wenzel, 1953, *Lamtostyla* Buitkamp, 1977, and *Perisincirra* Jankowski, 1978. Later, Cladotrichidae was considered a junior synonym of Kahliellidae Tuffrau, 1979 by several authors (Jankowski 2007; Lynn 2008; Lynn and Small 2002; Tuffrau and Fleury 1994). By contrast, Berger (2011) preliminarily synonymized Cladotrichidae with Gonostomatidae Small and Lynn 1985 because the oral apparatus of *Cladotricha* very closely resembles that of *Gonostomum* Sterki, 1878.

*Cladotricha* has a gonostomatid oral apparatus, usually one short and one or two long frontoventral rows, and two marginal rows. Postoral and pretransverse ventral and transverse cirri are lacking. The dorsal kinety pattern is simple, that is, consists of three bipolar bristle rows each of them bearing a caudal cirrus. All species described so far have been found in saline habitats (Berger 2011). Recently, Foissner (2012) discovered *Schmidingerothrix extraordinaria*, a *Cladotricha*-like species with an extremely reduced infraciliature, that is, besides several cirral groups (middle and right frontal cirrus; buccal cirrus; postoral, transverse, and caudal cirri) even the paroral and the dorsal bristles have been lost. Since no molecular data are available for this species from a highly saline soil in Namibia, the position of the monotypic Schmidingerotrichidae Foissner 2012 remains unknown in molecular trees. In May 2006, we discovered a *Cladotricha*-like hypotrich with a partially strongly reduced ciliature in a hypersaline pond near the coastal waters off Qingdao, China. Fortunately, we found some dividers and could sequence its small-subunit rRNA gene (SSU rDNA). Therefore, it is possible to estimate the phylogenetic position of a “cladotrichid” for the first time with a molecular marker.

## Materials and methods

### Sample site and cultivation

*Paracladotricha salina* n. sp. has been found in an abandoned offshore mollusc-farming pond of the Red island off the mouth area of Jiaozhou Bay of Qingdao (N 36°04′, E 120°18′), China, on 23 May 2006, when the water temperature was about 17 °C, the pH 8.0, and salinity 80‰. Glass slides were used as artificial substrate to collect ciliates. Briefly, the slides were fixed to a frame and immersed in the ponds to a depth of 1 m for 7–10 d to allow colonization. The slides were transferred to Petri dishes containing water from the sampling site. Isolated specimens were maintained at 20 °C and a salinity of about 50‰ and squeezed rice grains were added to enrich the bacterial food (Chen et al. 2013a,b2013b; Pan et al. 2012). Unfortunately, we could not establish a clone of *P. salina* and therefore we cannot be 100% sure that the specimens used for the morphological studies and molecular analyses belong to the same species. This is a general problem, which is true of very many ciliate species. However, when the procedures are done by experienced taxonomists the probability is very low to deal with two species. After the identification based on several cells, five identical cells were isolated from the same sample to extract the genomic DNA.

### Morphological and morphogenetic methods

Living ciliates were examined using bright field and differential interference contrast microscopy. The Protargol method was applied to reveal the infraciliature and nuclear apparatus (Wilbert 1975). Measurements and counts on impregnated specimens were performed at a magnification of 1,250X. Drawings of the infraciliature were made with the help of a camera lucida. To illustrate the changes occurring during morphogenesis, old (parental) ciliary structures are depicted by contour whereas new ones are shaded black (Chen et al. 2013c; Shao et al. 2013). General terminology is mainly according to Lynn (2008), for terms specific for hypotrichs (e.g., DE-value, gonostomatids oral apparatus) see Berger (1999, 2006, 2008, 2011) and Foissner and Al-Rasheid (2006). For the designation of the frontal–ventral cirri and anlagen, the numbering system established by Wallengren (1900) is used (for details, see Berger 1999, 2008).

### Extraction, PCR amplification, and sequencing of DNA

Extraction of genomic DNA, amplification of SSU rRNA genes by polymerase chain reaction (PCR), cloning, and sequencing were performed according to Huang et al. (2012) and Yi and Song (2011). Amplification of the SSU rRNA gene was performed with the universal primers Eukaryotic A (5′-AAC CTG GTT GAT CCT GCC AGT-3′) and Eukaryotic B (5′-TGA TCC TTC TGC AGG TTC ACC TAC-3′) (Medlin et al. 1988).

### Sequence availability and phylogenetic analyses

Analyses were based upon a dataset comprising the SSU rRNA sequence of *P. salina* n. sp. and 60 SSU rRNA sequences of spirotrichous ciliates from GenBank. The accession numbers are provided after the species names in the phylogenetic tree (Fig. 4). Sequences were aligned using ClustalW implemented in BioEdit 7.0.0 (Hall 1999), and were further modified manually using SeaView (Gouy et al. 2010) to exclude the highly variable and ambiguous regions which are difficult to align. The final alignment used for phylogenetic analyses includes 1,741 positions. Bayesian inference (BI) and maximum likelihood (ML) methods were used to construct the phylogenetic trees. Model selecting and software used in the analyses are according to Pan et al. (2013). Briefly, BI analyses were performed with MrBayes 3.1.2 (Ronquist and Huelsenbeck 2003) under a GTR+I+Γ model. Markov chain Monte Carlo simulations were then run with two sets of four chains using the default settings, with a sampling frequency of 0.01. The chain lengths were 1,000,000 generations, and 25% of generations were discarded as burn-in. The remainder was used to generate consensus trees and to calculate the posterior probabilities of all branches using a majority-rule consensus approach. The ML tree was constructed with the PhyML V2.4.4 (Guindon and Gascuel 2003) program under the model GTR+I+Γ. The reliability of internal branches was assessed using a nonparametric bootstrap method with 1,000 replicates. Approximately unbiased (AU) tests (Shimodaira 2002) were performed to compare the ML tree with the hypothesis that the gonostomatids form a monophyletic group. RAxML (Stamatakis et al. 2008) was used to search for the best fit ML tree that fits the relevant constraint, starting from 20 distinct randomized maximum parsimony starting trees under the model GTR+I+Γ. Considering the deviation between the two programs (PhyML and RAxML), an unconstrained RAxML analyses were also performed to search for the optimal RAxML tree, using the same model and parameter settings. The constraint tree was then compared to both the optimal RAxML and PhyML trees. The site-wise likelihoods were calculated for each tree topology with RAxML. AU tests were then performed using CONSEL 0.1 (Shimodaira and Hasegawa 2001).

## Results

### Morphology of *Paracladotricha salina* n. g., n. sp. (Table 1; Fig. 1, 3A–G, L)

**Figure 1:**
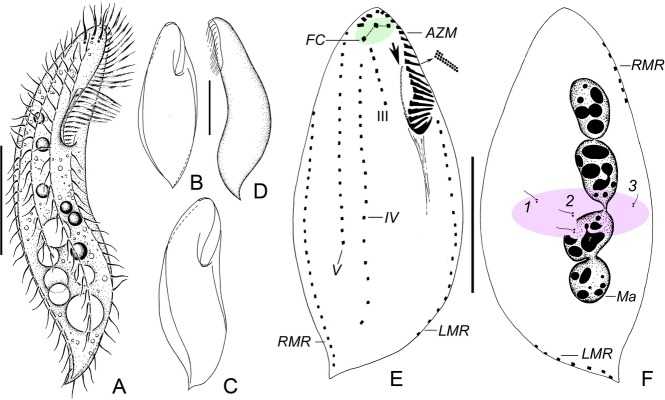
Morphology of *Paracladotricha salina* n. g., n. sp. from life (A–D) and after protargol impregnation (E, F). **A.** Ventral view of a representative individual; note sigmoidal body shape. **B, C.** Ventral views showing variability in body shape. **D.** Left lateral view showing dorsoventral flattening. **E, F.** Infraciliature of ventral and dorsal side and macronuclear apparatus of holotype specimen. Note that adoral membranelles are composed of only three rows of cilia. Arrow in (E) marks endoral. AZM = adoral zone of membranelles; FC = frontal cirri; LMR = left marginal row; Ma = macronuclear nodules; RMR = right marginal row; III, IV, V = frontoventral rows III, IV, V; 1–3 = dorsal kineties. Scale bars 30 μm.

Body size rather variable, namely 50–120 × 20–35 μm in vivo, ratio of length to width about 2.5–4.5:1 (five cells observed). Body fusiform, sigmoidal, and slightly twisted about main body axis; left and right margin almost parallel in middle portion; anterior end narrowly rounded, rear one acuminate. Cells not distinctly flattened dorsoventrally, that is, almost circular in cross-section (Fig. 1A–D). Cytoplasm hyaline and colorless, usually contains many lipid droplets about 2–3 μm across and several food vacuoles containing, inter alia, algae (Fig. 3A–F). Pellicle thin and soft, body shape thus rather variable; however, cells not distinctly contractile. Contractile vacuole not observed, distinct cortical granules lacking. Macronuclear apparatus moniliform, that is, three or four globular or ellipsoidal nodules arranged behind one another somewhat right of cell midline (Fig. 1F, 3H). Micronuclei not recognizable in nondividers with the method used. Cells often floating motionless for more than 30 s, sometimes slowly crawling on substrate; when disturbed, swimming straight forward by rotation about main body axis.

**Table 1:** Morphometric characterization of *Paracladotricha salina* n. g., n. sp

Character^a^	HT	Min	Max	Mean	SD	*n*
Body, length	83	72	106	92.2	8.9	16
Body, width	32	29	48	37.1	4.8	16
Adoral zone, length	28	27	42	34.4	4.3	16
Adoral membranelles, no.	23	20	29	23.2	2.1	16
Frontal cirri, no.	3	3	3	3.0	0	16
Left marginal cirri, no.	25	13	25	18.3	3.3	16
Right marginal cirri, no.	31	20	31	25.8	3.4	16
FVR III, no. of cirri	6	4	7	5.7	1.1	16
FVR IV, no. of cirri	18	12	25	17.7	3.8	16
FVR V, no. of cirri	18	10	18	14.1	2.9	16
Dorsal kineties, no.	3	3	3	3.0	0	16
Dorsal kinety 1, no. of bristles	1	1	2	1.3	0.5	9
Macronuclear nodules, no.	4	3	4	3.9	0.3	16

a All data based on protargol-impregnated specimens. Measurements in μm.

FVR = frontoventral row; HT = holotype specimen (also included in the sample *n*); Max = maximum; Mean = arithmetic mean; Min = minimum; *n* = number of cells measured; SD = standard deviation.

Adoral zone basically in *Gonostomum* pattern (Berger 2011), occupies 30–40% of body length, composed of 23 membranelles on average, distalmost three membranelles notably offset (Fig. 3L). Distal end extends to 6% of body length in holotype, that is, DE-value 0.18 (Fig. 1E). Bases of largest membranelles about 8 μm wide, cilia up to 15 μm long in vivo; individual membranelles composed of only two long and one very short row of basal bodies. Buccal cavity inconspicuous because narrow and slit-like. Only one undulating membrane present, likely the endoral (see Discussion), composed of monokinetids, almost straight, commences at about 16% of body length in holotype, terminates close to rear end of adoral zone (Fig. 1E). Pharyngeal fibers extend posteriorly. Three slightly enlarged frontal cirri, left and middle one at level of distal end of adoral zone, right one behind distal end of zone, cilia about 12 μm long in vivo. Buccal cirrus lacking. Frontoventral cirral row III behind right frontal cirrus, composed of 4–7 cirri, terminates at 27% of body length in holotype specimen (Fig. 1E). Two long frontoventral rows between frontoventral row III and right marginal row; left one (termed frontoventral row IV from now on) composed of 12–25 cirri, somewhat shortened anteriorly (commences at 15% of body length in holotype) and posteriorly (85%); right one (frontoventral row V) consisting of 10–18 cirri, commences about at level of right frontal cirrus (8%), extends to about 65% of cell length in holotype. Left marginal row composed of 13–25 cirri, commences at 30% of body length in holotype, that is, slightly ahead of level of buccal vertex, extends dorsomarginally in rear body portion (Fig. 1E, F). Right marginal row made of 20–31 cirri, commences dorsomarginally about at level of right frontal cirrus. Both marginal rows terminate close to posterior body end that is, only inconspicuously separated. Isolated postoral and pretransverse ventral cirri as well as transverse cirri lacking. Bases of all cirri, except frontal one, likely composed of four kinetosomes; cilia about 8–10 μm long (Fig. 1E). Invariably three very strongly reduced dorsal kineties, that is, each row composed of only one or two dikinetids about in mid-body. Dorsal cilia 4–5 μm long in vivo (Fig. 1F). Caudal cirri lacking.

### Morphogenesis of *P. salina* n. g., n. sp. (Fig. 2, 3H–K)

We found some dividers so that we can provide important details on this part of the life cycle. The frontal-ventral anlagen are designated I–V, although it is not certain that anlage VI, which forms the right transverse cirrus and the frontoterminal cirri in 18-cirri hypotrichs (e.g., *Gonostomum affine*), is lacking.

**Figure 2:**
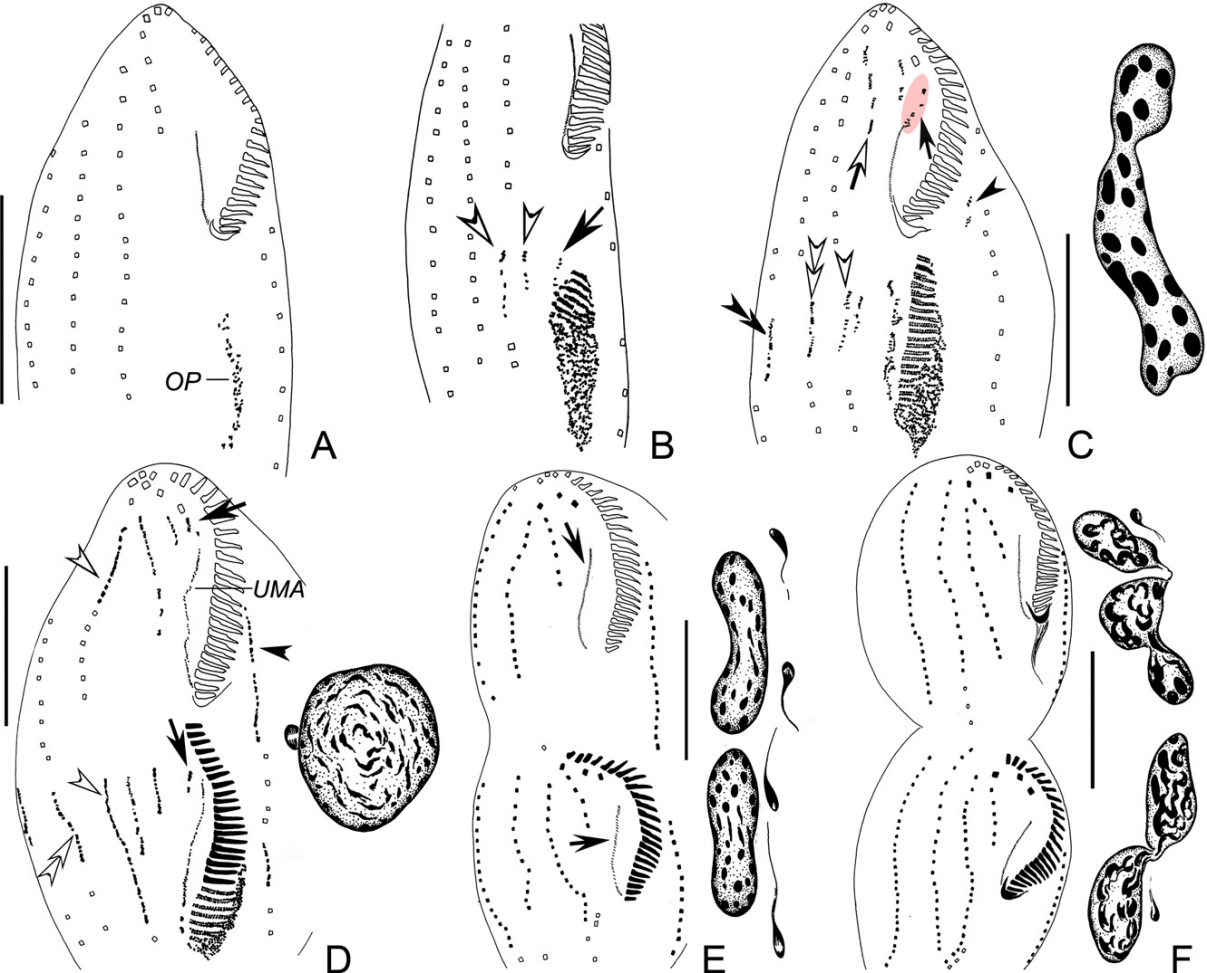
Morphogenesis of ventral side of *Paracladotricha salina* n. g., n. sp. after protargol impregnation. **A.** Very early divider. **B.** Early divider, arrow indicates the undulating membrane anlage splitting anteriorly from the oral primordium, and white arrowheads mark the newly formed anlagen III and IV of the opisthe. **C.** Early to middle divider; double-arrowhead denotes right marginal anlage of opisthe, black arrowhead marks left marginal row anlage of proter; white arrowhead and white double-arrowhead mark anlagen IV and V of opisthe, respectively; black arrow depicts disaggregating parental endoral (anlage I); anlage III of proter originates by complete modification of parental row III (white arrow); left of it, anlage II is formed de novo. Insert: the individual macronuclear nodules fused. **D.** Middle divider; white arrowheads mark the anlagen IV in parental row IV; white double-arrowhead depicts anlage V of opisthe in parental row, arrows mark left frontal cirrus which is formed from anlage of endoral in both filial products; arrowheads indicate left marginal row anlagen. Insert: fused, globular macronucleus and micronucleus. **E, F.** Dividers in late stages showing the formation of the cirral pattern. Arrows mark the newly formed endorals. Inserts: division of nuclear apparatus. OP = oral primordium; UMA = undulating membrane anlage of proter. Scale bars 30 μm.

**Figure 3:**
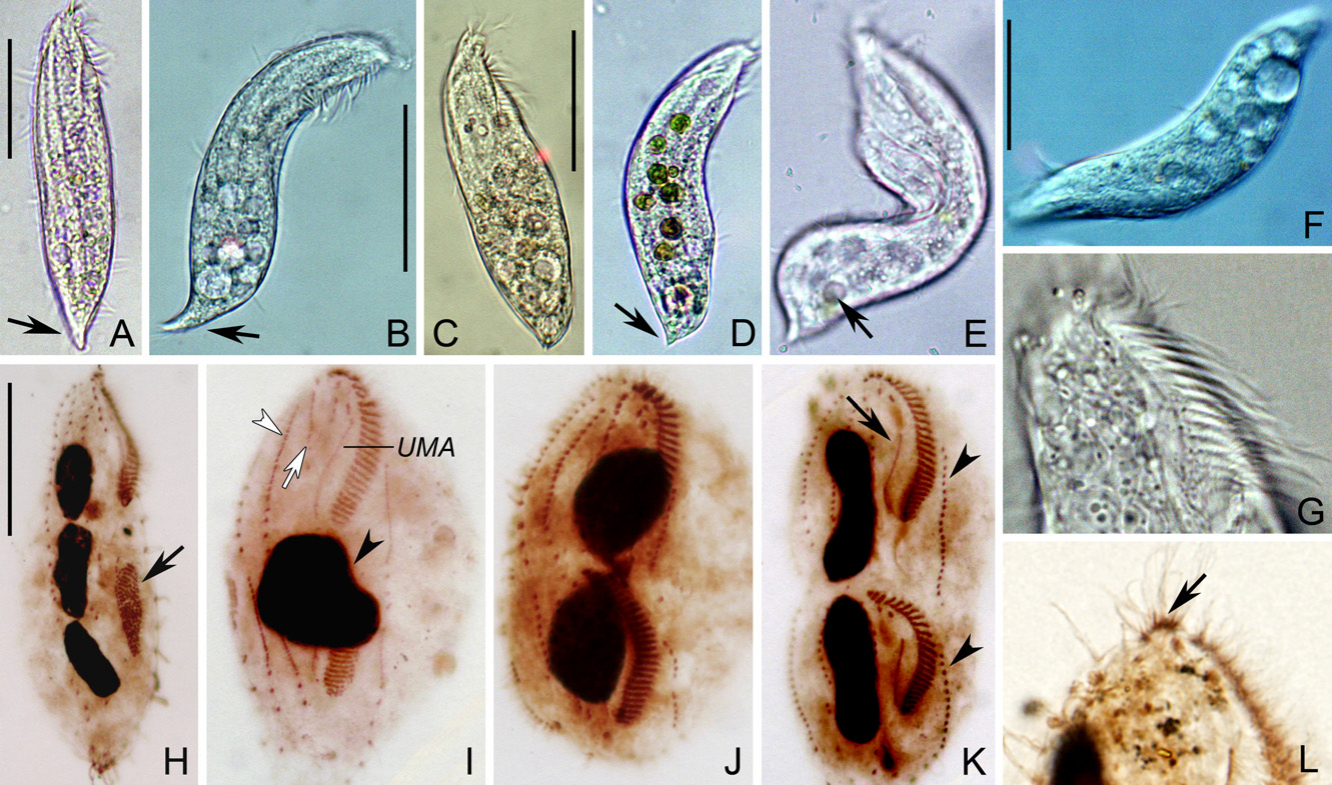
Microphotographs of *Paracladotricha salina* n. g., n. sp. from life during interphase (A–D, G, bright field; E, F, differential interference contrast) and after protargol impregnation during binary division (H–L). **A, B.** Ventral views of typical individuals; note acuminate rear end of cell (arrows). **C-F.** Various views showing, inter alia, different body shapes, cytoplasmic inclusions, acuminate rear end of cell (arrow in D), and food vacuole (arrow in E). **G.** Oral region showing middle and proximal portion of gonostomatid adoral zone. **H.** Ventral view of an early divider with oral primordium (arrow) in opisthe. **I.** Ventral view of a middle divider. Arrowhead marks fused macronucleus, white arrow and white arrowhead mark cirral anlagen III and IV in proter, respectively. **J, K.** Ventral views of late dividers showing development of new cirri and macronuclear nodules. Arrow and arrowheads in (K) mark new endoral of proter and new left marginal rows respectively. **L.** Anterior portion of ventral side showing the distalmost three membranelles notably offset (arrow). UMA = undulating membrane anlage of proter. Scale bars 30 μm.

#### Oral apparatus

Division commences with the proliferation of basal bodies in the postoral region (Fig. 2A). The oral primordium becomes larger and the anterior portion commences with the differentiation of adoral membranelles for the opisthe (Fig. 2B). Right of the anterior end of the oral primordium, the undulating membrane anlage is generated. The parental undulating membrane begins to disorganize into an anlage while the undulating membrane anlage in the opisthe becomes longer due to proliferation of basal bodies (Fig. 2C). Simultaneously, the formation of new membranelles proceeds from the front backwards. In later stages, the anterior portion of the new adoral zone in the opisthe bends to the right. Meanwhile, anlage I gives rise to the new endoral in both filial products (Fig. 2E, F). Obviously, the parental adoral zone is taken over by the proter.

#### Frontoventral ciliature and marginal rows

The anlagen I–V of the proter and the opisthe are formed independently from each other, that is, no primary primordia occur (Fig. 2B–E). In the opisthe, two streaks are formed to the right of the anterior end of the oral primordium and undulating membrane anlage (Fig. 2B). The left one obviously originates de novo and eventually forms, very probably, anlage III. The right one originates within the parental frontoventral row IV and forms the anlage for the new frontoventral row IV (Fig. 2B). Later, in the proter a small streak is formed de novo behind the middle frontal cirrus; it becomes anlage II. By contrast, anlage III develops from the dedifferentiated cirri of the parental frontoventral row III (Fig. 2C). In the opisthe, a streak develops in the parental frontoventral row V to form the anlage for this row (Fig. 2C). Simultaneously, the right marginal row primordium of the opisthe and the left marginal row anlage of the proter begin to differentiate within the parental rows. In a somewhat later stage, the left frontal cirrus (I/1) originates, as is usual, from the anterior end of the undulating membrane anlagen (Fig. 2D). Anlage II of the opisthe appears, obviously de novo due to the lack of parental structures in this area, between the left frontal cirrus and anlage III. In the proter, anlage IV is formed, as in the opisthe, within the parental frontoventral row IV (Fig. 2D). Very likely, anlage V of the proter originates, like the anlage V of the opisthe, within the parental frontoventral row V, that is, five frontoventral cirri anlagen are now present in both filial products (Fig. 2D). In late and very late dividers, the final number of cirri is formed within the new frontoventral rows, and the frontal cirri and rows start to migrate to their final positions (Fig. 2E, F). The marginal rows develop within the parental rows. Obviously, no parental cirri are retained after division.

#### Dorsal ciliature

We could not clarify the formation of the strongly reduced dorsal ciliature due to the lack of useable dividers.

#### Nuclear apparatus

The division of the nuclear apparatus proceeds as in most other hypotrichs. The micronuclei divide mitotically, whereas the fused macronucleus makes some successive amitotic divisions to produce the species-specific pattern (Fig. 2C–F, 3H–K).

### Phylogenetic analyses based on SSU rRNA gene sequences (Fig. 4)

**Figure 4:**
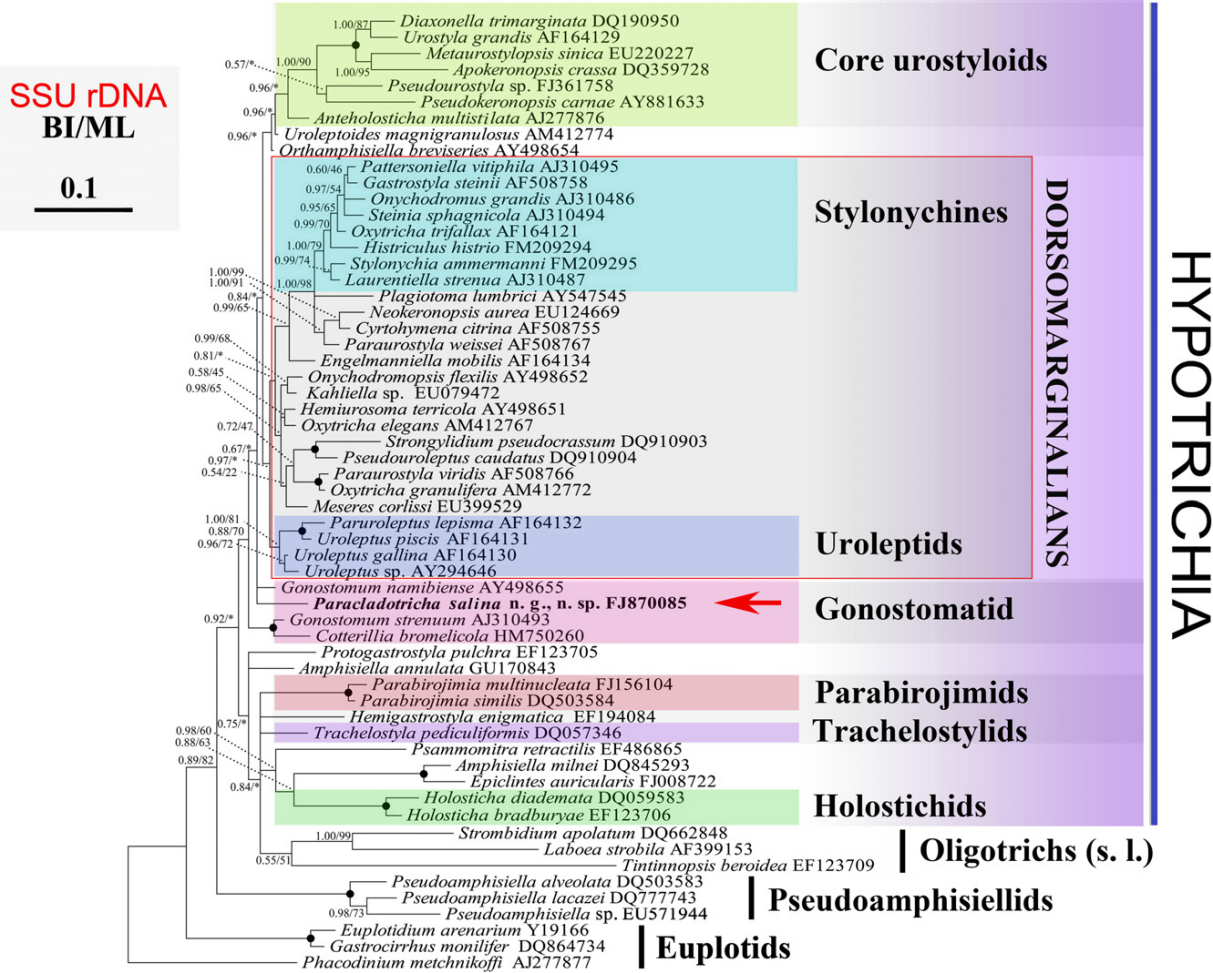
Bayesian Inference (BI) tree of spirotrich SSU-rDNA sequences to estimate the phylogenetic position of *Paracladotricha salina* (bold). Numbers at nodes are the posterior probabilities from Bayesian Inference (BI) and the bootstrap values from Maximum likelihood (ML) algorithm. Nodes receiving maximal support (100 ML, 1.00 BI) are indicated by solid circles. Accession numbers are provided after species names. Scale bar corresponds to 0.1 expected substitutions per site. Asterisks at nodes indicate bootstrap values < 50%/0.50 and disagreement between the two methods. Note that only higher taxa are indicated which are relatively well-defined. The Hypotrichia and the gonostomatids are nonmonophyletic in the present tree, a result which should not be over-interpreted. *Oxytricha trifallax* is likely identical, or at least very similar to *Sterkiella histriomuscorum* (Foissner and Berger 1999); at least it is not an *Oxytricha* because it clusters within the stylonychines (see also Zoller et al. 2012).

The SSU rRNA sequence of *P. salina* (NCBI accession number FJ870085) has a length of 1,769 bp and a GC content of 45.05%. All trees constructed with BI and ML methods have a similar topology and thus only the BI tree is shown (Fig. 4). *Paracladotricha salina* branches off, together with *Gonostomum namibiense* Foissner et al., 2002, rather near the base of the Hypotrichia tree, that is, it is sister to a large group mainly composed of the dorsomarginalians and the (core) urostyloids (Fig. 4). Shortly before, a small cluster composed of *Gonostomum strenuum* (Engelmann, 1862) and *Cotterillia bromelicola* Foissner and Stoeck 2011 branches off, however, with low support in the BI tree (0.67 BI). The result of the AU test based on the ML analyses did not reject the hypothesis that all gonostomatids group together: AU number for constraint tree is 0.166, while those for the optimal RAxML and PhyML trees are 0.829 and 0.299, respectively.

## Discussion

### Comparison of *P. salina* n. g., n. sp. with related and similar taxa

The gonostomatid oral apparatus, the frontoventral rows, and the highly saline habitat indicate that our population is closely related with *Cladotricha* species (for review, see Berger 2011). Unfortunately, *Cladotricha* is an ill-defined genus because the type species (*C. koltzowii*) was never seriously described in all details (Berger 2011). One major problem is that the dorsal kinety pattern is not known (Borror and Evans 1979; Gaievskaïa 1925; Ruinen 1938). The other species assigned to *Cladotricha* have a ventral infraciliature which is very similar to that of our population, namely, three frontal cirri, a short frontoventral row III, one or two long frontoventral rows, and one left and one right marginal row. Postoral ventral cirri and transverse cirri are lacking as in our population (Blatterer and Foissner 1988; Borror and Evans 1979; Ruinen 1938; Wilbert 1995). The dorsal infraciliature of these *Cladotricha* species is as in *Gonostomum*, that is, three bipolar kineties each bearing a caudal cirrus. Berger (2006, 2008, 2011) hypothesized this simple dorsal kinety pattern, which lacks fragmentation and dorsomarginal rows, as part of the ground pattern of the Hypotrichia.

In spite of the similarities, our population differs distinctly from *Cladotricha* species and all other species described so far in several features, inter alia, by the lack of the buccal cirrus and the paroral, and a highly reduced dorsal infraciliature. A loss of the buccal cirrus is described only for *S. extraordinaria* (Foissner 2012) and *C. koltzowii* sensu Borror and Evans (1979). However, *C. koltzowii* has the ordinary two undulating membranes while our population has lost the paroral. Considering that even minor differences in the shape of the paroral or endoral are used as generic features (e.g., *Notohymena* Blatterer and Foissner, 1988), the separation of our population from *Cladotricha* at genus level seems justified (Berger 1999).

*Schmidingerothrix* is likely the hypotrich with the most strongly reduced infraciliature described so far. Moreover, it lacks, like *P. salina*, the paroral, which is usually short and composed of a rather low number of relatively loosely arranged cilia in hypotrichs with a gonostomatid oral apparatus (e.g., Berger 2011; Foissner and Stoeck 2011). Perhaps the most interesting feature of our population is the very strongly reduced dorsal ciliature: only one or two dikinetids are still present per kinety about in mid-body (Fig. 1F). This reduction is also reminiscent of *S. extraordinaria* where, however, the dorsal infraciliature fully disappeared. Further morphological differences between these two taxa consist in the number of frontal cirri (three in *P. salina* vs. only one in *S. extraordinaria*) and long frontoventral rows (two vs. none), the cortical granulation (lacking vs. present), and the adoral zone (continuous vs. bipartite). In addition, *P. salina* and *S. extraordinaria* live in different habitats and biogeographic regions, namely a hypersaline offshore pond in China against a highly saline soil in the Etosha National Park in Namibia (Foissner 2012). These comparisons demonstrate that our population belongs to a new species which is, in addition, a representative of a new genus: *P. salina* n. g., n. sp. (for characterization, see below).

### Phylogenetic position of *Paracladotricha* n. g.

Phylogenetic analyses based on gene sequence data place gonostomatid hypotrichs outside the Dorsomarginalia Berger, 2006 (e.g., Affa'a et al. 2004; Foissner and Stoeck 2011; Jiang et al. 2013; Paiva et al. 2009), that is, they usually branch off rather early. Mostly, they even branch off earlier than the (core) urostyloids, that is, near the base of the Hypotrichia tree (for review, see Berger 2011).

So far, only three species with a typical gonostomatid oral apparatus have been studied using molecular phylogenetic methods, namely, *G. strenuum*, *G. namibiense*, and *Cotterillia bromelicola*. The gene sequence analyses by Foissner and Stoeck (2011) supported the basically morphology-based classification of *Cotterillia* in the gonostomatids, indicating that this easily recognizable morphological feature is a good phylogenetic marker, however, only in combination with the dorsal kinety pattern. *Kahliella* Corliss, 1960, which has a similar oral apparatus, has a dorsomarginal kinety, indicating that gonostomatids and kahliellids are not closely related (Berger 2011; Eigner 1995; Vd'ačný et al. 2010). *Paracladotricha salina* is, according to the BI and ML method (Fig. 4), a further species belonging to the gonostomatids, although the gene sequence analyses did not result in a monophyletic group composed of these four species only. However, considering the weak support values on the tree nodes and a rather high *p* value (0.214) from the AU test results, we could not reject a possibility of the monophyly of the gonostomatids. Given that the incapability of the SSU rRNA gene data on resolving the relationships among the gonostomatids, the assignment of this new genus relies more on the morphological data.

**Figure 5:**
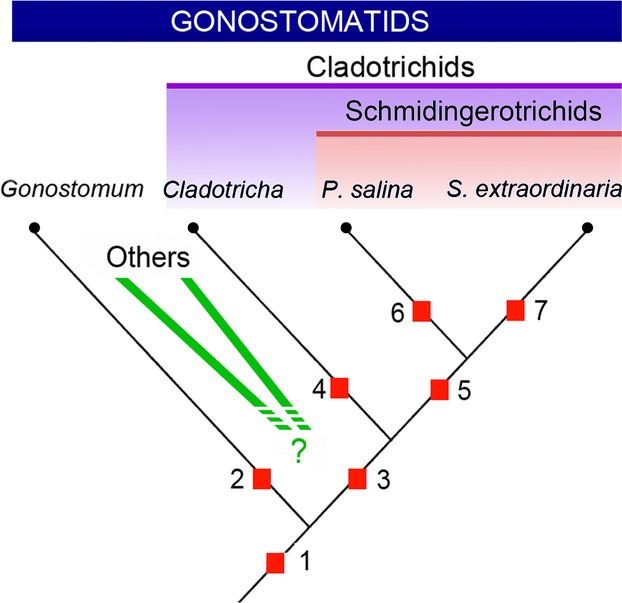
First vague hypothesis about the phylogenetic relationship between taxa with a gonostomatid oral apparatus. Main apomorphies (squares 1–7): 1 = gonostomatid oral apparatus; 2 = postoral ventral cirri displaced anteriad (?); 3 = five frontal-ventral anlagen; frontoventral rows present; postoral ventral cirri lost; transverse cirri lost; saline habitat; 4 = no apomorphy known at present state of knowledge; 5 = buccal cirrus lost (according to Borror and Evans 1979, the type species *Cladotricha koltzowii* also lacks a buccal cirrus, however, detailed redescription needed; other species assigned to this genus, have this cirral group); paroral lost; medium-length row of adoral membranelles lost; dorsal kineties strongly reduced; caudal cirri lost; 6 = cortical granules lost; 7 = left and middle frontal cirrus lost; long frontoventral rows lost; dorsal kineties lost. The “others” comprise taxa like *Paragonostomum*, *Wallackia*, *Neowallackia*, *Cotterillia*; their origin remains more obscure than that of the genera and species mentioned in the tree. For the ground pattern of the Hypotrichia and Gonostomatidae see Berger (2008, 2011).

We preliminarily assign *Paracladotricha* to the so far monotypic Schmidingerotrichidae because both *Schmidingerothrix* and *Paracladotricha* lack the paroral and a buccal cirrus, have adoral membranelles composed of three rows only, have a very strongly reduced dorsal infraciliature (lacking, respectively, composed of very few dikinetids), and are likely confined to highly saline habitats (Fig. 5). However, since no gene sequence data are available for *S. extraordinaria*, we do not have molecular biological evidence for this classification.

*Cladotricha*, which also closely resembles *Paracladotricha*, is still ill-defined due to a lack of a detailed redescription, neotypification, and gene sequence data of the type species, and *Gonostomum affine* (Stein, 1859), type of *Gonostomum* Sterki, 1878, is not yet analyzed with molecular methods. These shortcomings prevent a serious, detailed discussion of the validity and phylogenetic relationships of the Gonostomatidae, the Cladotrichidae, and the Schmidingerotrichidae. The discovery of further species belonging to the Gonostomatidae, Cladotrichidae (classified as synonym of the Gonostomatidae by Berger 2011), and schmidingerotrichids (monotypic and thus basically redundant without *Paracladotricha*) will perhaps show that all three taxa are valid and form a monophyletic group (the gonostomatids) with the gonostomatid oral apparatus as main morphological apomorphy (Fig. 5). Further, it is conceivable that *Cladotricha*—or the cladotrichids sensu stricto because *Cladotricha* is perhaps not monophyletic (see below)—and the schmidingerotrichids form a subgroup (the cladotrichids) within the supposed monophyletic group Gonostomatidae. Possible morphological apomorphies of the cladotrichids are (i) the long frontoventral rows (still present in *P. salina*, but already lost in *S. extraordinaria*), (ii) the loss of some cirral groups (transverse cirri, postoral ventral cirri), and (iii) in total not six, but only five anlagen (which anlage was conserved [V or VI] is difficult to estimate). The colonization of highly saline habitats could be an ecological novelty (Fig. 5).

We also hypothesize that *Paracladotricha* and *Schmidingerothrix* are a subgroup of the cladotrichids with a very strongly reduced dorsal ciliature and a further loss of cirral groups (caudal cirri, buccal cirrus) and the paroral. These two genera (both are monotypic so far) form the schmidingerotrichids. This is a first vague hypothesis to unravel the relationships within the gonostomatids. *Paracladotricha* and *Schmidingerothrix* are characterized, inter alia, by the lack of the paroral (Fig. 1E; Foissner 2012). In hypotrichs with a gonostomatid oral apparatus, the paroral is generally composed of relatively few (compared to other hypotrichs), more or less widely spaced cilia. Some species assigned to the gonostomatids in the review by Berger (2011), already show a trend to reduce the number of the paroral cilia further to only 1–5 (e.g., *Paragonostomum minuta* Kamra et al., 2008; *Neowallackia* spp.). In *Cladotricha*, *C. australis* Blatterer and Foissner, 1988 and *C. halophila* Wilbert, 1995 have a very low number of paroral cilia, while Borror and Evans (1979) described two rather long undulating membranes for the type species *C. koltzowii* which was originally discovered in Crimea (Gaievskaïa 1925). However, since the identification of the American population (Borror and Evans 1979) is not quite certain and since molecular data are lacking we refrain from a splitting of *Cladotricha* based on this feature.

## Taxonomic summary

Ciliophora Doflein, 1901

Spirotrichea Bütschli, 1889

Hypotrichia Stein, 1859

### *Paracladotricha* n.g. (Table 1; Fig. 1, 3A–G, L)

**Diagnosis.** Hypotrichia with gonostomatid adoral zone, three frontal cirri, and one short and two long frontoventral rows, that is, lacking a paroral membrane and buccal, postoral, pretransverse ventral, and transverse cirri; one right and one left marginal row. Dorsal ciliature not lacking, but kineties 1–3 highly degraded; caudal cirri absent. Highly saline habitats (waters?).

**Type species.** *Paracladotricha salina* n. sp.

**Etymology.** *Paracladotricha* is a composite of the Greek prefix *para-* (related; Werner 1972) and the genus-group name *Cladotricha* (see Berger 2011 for derivation), alluding to the fact that this new genus is likely (closely?) related with *Cladotricha*. Like *Cladotricha* of feminine gender (Aescht 2001).

**Species included.** *Paracladotricha salina* n. sp.

### *Paracladotricha salina* n.sp.

**Diagnosis.** Slender, transparent *Paracladotricha* with narrowly rounded anterior and pointed posterior end. Body size in vivo about 50–120 × 20–35 μm. Four macronuclear nodules. Adoral zone about 37% of body length and composed of 23 membranelles on average. Three frontoventral rows, composed of 4–7 cirri (row III, distinctly shorter than adoral zone), 12–25 (row IV, almost bipolar), and 10–18 (row V, distinctly shortened posteriorly). Right marginal row consists of an average of 26 cirri, left of 18. Dorsal kineties 1–3 composed of only one or two basal body pairs each in about mid-body.

**Type locality.** Abandoned offshore mollusc-farming pond of the Red island off the mouth area of the Jiaozhou Bay of Qingdao (N 36°04′, E 120°18′), China (further details, see Materials and methods).

**Etymology.** The Latin adjective *salin·us*,*-a*,*-um* (m, f, n; of salt, saline; Werner 1972) refers to the habitat where the species was discovered.

**Type material**. The protargol slide (registration number LLQ-2006-05-23-2C1) containing the holotype specimen (Table 1, Fig. 1E, F) and a paratype slide (registration number LLQ-2006-05-23-2C2) with about 20 protargol-impregnated specimens have been deposited in the Laboratory of Protozoology, Ocean University of China (OUC), Qingdao, China. The SSU rRNA sequence is deposited at GenBank, accession number FJ870085.
